# Longitudinal associations of health-related behavior patterns in adolescence with change of weight status and self-rated health over a period of 6 years: results of the MoMo longitudinal study

**DOI:** 10.1186/1471-2431-14-242

**Published:** 2014-09-30

**Authors:** Sarah Spengler, Filip Mess, Eliane Schmocker, Alexander Woll

**Affiliations:** University of Konstanz, Sports Science, Universitätsstraße 10, 78457 Konstanz, Germany; Karlsruhe Institute of Technology, Sports Science, Engler-Bunte-Ring 15, 76131 Karlsruhe, Germany

**Keywords:** Health-related behavior patterns, Lifestyle, Adolescents, Weight status, Subjective health, Longitudinal, Cluster analysis, Germany

## Abstract

**Background:**

Promoting a healthy lifestyle especially in adolescents is important because health-related behaviors adopted during adolescence most often track into adulthood. Longitudinal studies are necessary for identifying health-related risk groups of adolescents and defining target groups for health-promoting interventions. Multiple health behavior research may represent a useful approach towards a better understanding of the complexity of health-related behavior. The aim of this study was to examine the longitudinal association of health-related behavior patterns with change of weight status and self-rated health in adolescents in Germany.

**Methods:**

Within the framework of the longitudinal German Health Interview and Examination Survey for Children and Adolescents (KiGGS) and the Motorik-Modul (MoMo), four clusters of typical health-related behavior patterns of adolescents have been previously identified. Therefor the variables ‘physical activity’, ‘media use’ and ‘healthy nutrition’ were included. In the current study longitudinal change of objectively measured weight status (N = 556) and self-rated health (N = 953) in the four clusters was examined. Statistical analyses comprised T-tests for paired samples, McNemar tests, multinomial logistic regression analysis and two-way ANOVA with repeated measures.

**Results:**

The prevalence of overweight increased in all four clusters. The health-related behavior pattern of low activity level with high media use and low diet quality had the strongest increase in prevalence of overweight, while the smallest and non-significant increase was found with the behavior pattern of a high physical activity level and average media use and diet quality. Only some significant relationships between health-related behaviour patterns and change in self-rated health were observed.

**Conclusions:**

High-risk patterns of health-related behavior were identified. Further, cumulative as well as compensatory effects of different health-related behaviors on each other were found. The information gained in this study contributes to a better understanding of the complexity of health-related behavior and its impact on health parameters and may facilitate the development of targeted prevention programs.

## Background

Health-related behaviors such as activity level and dietary habits have been recognized as key aspects of lifestyle that influence the risk for chronic diseases including obesity, cardiovascular disease and depression [1–3]. These behaviors are most often adopted in adolescence and track into adulthood [4–7]. Hence, promoting a healthy lifestyle systematically especially during adolescence is critical. However, primary prevention programs can only be implemented effectively if target groups are precisely defined and their behaviors and characteristics known. For instance, Carr stated that “there is a need for clearer definitions of target groups, their characteristics and particular needs” [8]. Multiple Health Behavior Research seems to be a promising approach for identifying target groups because it accounts for co-occurring or clustered health-related behaviors [9]. Clusters of behavioral patterns [10] represent combinations of behaviors that are more prevalent than single behaviors [11].

The approach of clustering health-related behaviors is based on the concept of health-related lifestyles [12, 13] which originates from the work of Max Weber (1922) [14]. Health-related lifestyles comprise a person’s health-related behaviors, health-related attitudes and their socio-structural context [12]. They are “collective patterns of health-related behavior based on choices from options available to people according to their life chances” [15]. According to this approach, health-related behavior patterns should first be identified and their socio-demographic correlates should be described. Second, the relationship between the identified behavior patterns and the development of health parameters should be evaluated. This approach would allow specifying high-risk groups of health limitations or chronic diseases in adulthood.

While measuring health in its entirety is a difficult challenge [16], it is possible to measure its indicators or risk factors such as weight status. Adolescent body mass index (BMI) has been shown to be associated with several health consequences [17] and even premature death (adjusted for adult BMI) [18, 19]. Another indicator of health receiving increasing attention [20] is general self-rated health (SRH). SRH is measured with a single item and expected to reflect the overall state of a person’s physical and mental health [20]. SRH has been identified as independent predictor of subsequent morbidity and mortality as shown in a review of 27 studies [21]. Further, SRH seems to be a predictor for future health expenditures [22] and is used to screen for high-risk groups [23].

In the past decade, a remarkable number of studies aimed to identify health-related behavior patterns in adolescents [24–36]. Many of these cross-sectional studies focused on energy balance-related behaviors [24, 27–36] and partly studied the association with overweight and, in one study, on cardiorespiratory fitness as a health parameter [34]. However, high-risk patterns can only be defined through longitudinal studies examining the development of health parameters in the different behavior patterns. To date, it is unclear which behavior patterns are in fact unhealthy and which are healthy.

Only few studies have examined the longitudinal association of multiple health-related behavior patterns with the development of a health parameter [24, 37–39]. For instance, Boone-Heinonen et al. [24] examined obesity-related behavior patterning in adolescents aged 11 to 21 years. Their analysis comprised 36 variables and revealed seven clusters for males and six for females. In their study, clusters were associated with incident obesity six years later in females but not in males. In females, the lowest incidence of obesity occurred in the “school clubs and sports” cluster. Gubbels et al. [37, 38] studied energy balance-related behavior patterns in 5-year-old children and identified four patterns: the “television-snacking” and the “sedentary-snacking” patterns were associated with longitudinal BMI development until the age of 8 years. All these studies [24, 37, 38] as well as the study of Landsberg et al. [39] observed weight status as health parameter. Results of Landsberg et al. [39] showed a lower incidence rate of obesity in their “high activity and medium-risk behavior” pattern in a regional sample of German adolescents. Overall, limited information on high-risk behavior patterns for overweight in Germany is available. Moreover, to our knowledge, to date the change of health parameters other than weight status have not been observed in the context of health-related behavior patterns.

The purpose of this study was to define high-risk patterns of health limitations and to obtain insights into the complex structure of multiple health behaviors. We examined the longitudinal association of health-related behavior patterns in adolescents in Germany with change in (a) weight status and (b) SRH and (c) stratified the data by sex and age group.

## Methods

### Data collection

**Figure 1:**
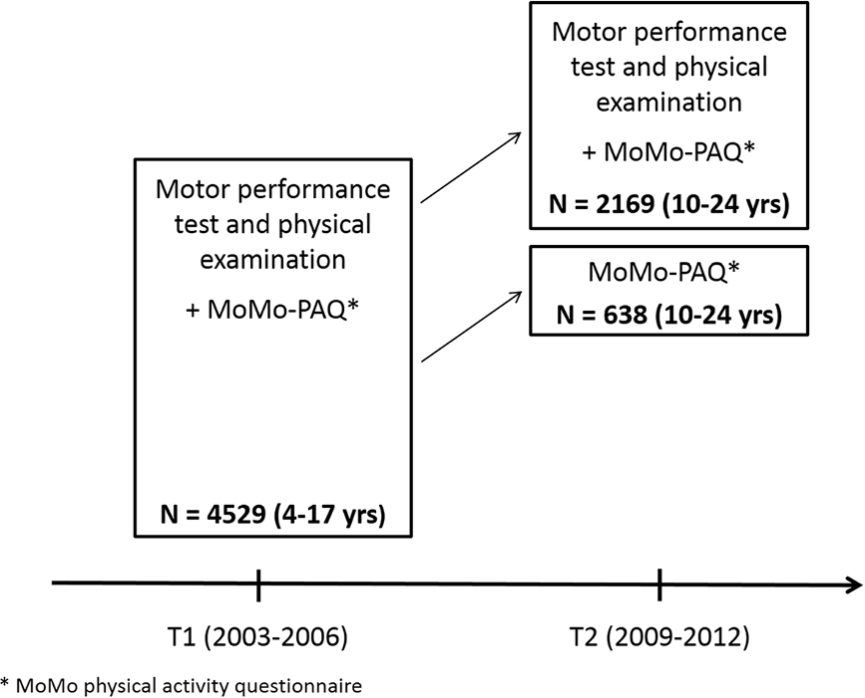
**Description of the MoMo longitudinal sample.**

The German Health Interview and Examination Survey for Children and Adolescents (KiGGS) [40] and the substudy ‘Motorik-Modul’ (MoMo) [41] are longitudinal studies that started in 2003. The goal of the KiGGS Survey is to collect nationwide representative data on health status of children and adolescents and to continuously monitor the development of health issues, health behavior and health risks in different population groups. KiGGS was approved by the Federal Office for Data Protection and by the ethics committee of the Charité University Hospital. The survey was conducted in accordance with the Declaration of Helsinki. The KiGGS baseline sampling (T1) was conducted by the Robert Koch-Institute (RKI) in Berlin and represents a nationwide cross-sectional survey on the health status of children and adolescents from 0 to 17 years of age [40]. For the representative subsample of the MoMo baseline (T1), comprehensive data on motor performance and physical activity of 4,529 children and adolescents aged between 4 and 17 years was collected between 2003 and 2006. Participants were recruited from the KiGGS sample allowing access to all parameters obtained in the KiGGS Survey. The first wave (T2) of the KiGGS Survey and MoMo Study took place between 2009 and 2012. Detailed descriptions of the longitudinal concept of the KiGGS Survey and the MoMo Study can be found in Hölling et al. [42] and in Wagner et al. [43], respectively. For the second sampling point of the MoMo Study, 2,807 longitudinal participants were recruited (response rate: 62%). Figure 1 illustrates the longitudinal sample of the MoMo Study. 2,169 participants attended the physical examination and completed the MoMo physical activity questionnaire at T2, and 638 participants only completed the questionnaire. For the current study, a subsample of adolescents between 11 and 17 years at T1 was used.

### Included variables

#### Health-related behavior patterns

In a previous study on T1 data of the MoMo Study (1,642 adolescents; 11–17 years) [29] four health-related behavior patterns could have been identified. In that study, participants completed a questionnaire assessing the amount and type of weekly physical activity in sports clubs and during leisure time, weekly use of television, computer and console games and the frequency and amount of food consumption.

For assessing physical activity the MoMo physical activity questionnaire (MoMo-PAQ) was used. Reliability (between k = 0.54 and k = 0.81, mean k = 0.66 (SD = 0.19) on item level) and validity (significant correlation between allover activity index and accelerometer Actigraph GT1X (Actigraph LLC, Pensacola, FL, USA) r = 0.29) of the questionnaire were similar to those of other questionnaires for measuring physical activity in adolescents [44]. Adolescents were asked about frequency, duration and type of their weekly habitual physical activity in the settings sports club and leisure time outside of sports clubs. Further, it was assessed in which months of a year the type of sport was performed. Adolescents could specify up to four different types of sports they perform in each setting [41]. From this data a physical activity index was derived: To include the intensity of different types of sports each reported sport was coded with the expended energy as metabolic equivalent of task (MET) per hour [45]. Subindices were calculated for every reported sport performed in sports club and in leisure time which indicate the METs expended per week with this specific type of physical activity (including frequency, duration and months in which this type of sport is performed). The maximally eight subindices were added to an overall activity index. Media use was assessed in the KiGGS Survey with a questionnaire asking the adolescents about the daily amount of time they spend on watching TV, using a computer and playing console games. According to Lampert [46] the answers were coded with the following values: ‘never’ = 0, ‘approx. 30 minutes’ = 0.5, ‘one to two hours’ = 1.5, ‘three to four hours’ = 3.5, ‘more than four hours’ = 5. The sum of these three variables represents the daily amount using these electronic media [46]. Food consumption was also assessed in the KiGGS Survey using a semi-quantitative food frequency questionnaire (FFQ) [47] covering 54 food items. The instrument was validated against a modified diet history instrument (DISHES) [48]. Ranking validity was fair to moderate, which is comparable to that of FFQs in the current literature (Spearman correlation coefficients from 0.22 to 0.69, most values 0.5 or higher) [49]. A healthy nutrition score (HuSKY) [50] was developed comparing adolescents’ food consumption with current recommendations for adolescents [51, 52]. The score reflects overall diet quality and ranges from 0 to 100 (=recommendations fully met). Details on the development of the HuSKY can be found in Kleiser et al. [50].

The three indices ‘physical activity’, ‘media use’ and ‘healthy nutrition’ had been included in the analysis and four stable clusters representing typical health-related behavior patterns had been identified (Table 1): Cluster 1 (16.2%) – high scores in physical activity index and average scores in media use index and healthy nutrition index; cluster 2 (34.3%) – high healthy nutrition score and below average scores in the other two indices; cluster 3 (18.6%) – low physical activity score, low healthy nutrition score and very high media use score; cluster 4 (30.9%) – below average scores on all three indices. The analysis of the current study is based on these clusters.

**Table 1:** **Mean (SD) values (z-scores) of the cluster solution, results of ANOVA** [29]

	Cluster 1	Cluster 2	Cluster 3	Cluster 4	F
N (%)	266 (16.2)	564 (34.3)	306 (18.6)	507 (30.9)	
HuSKY	0	0.94	−0.47	−0.76	605.68*
Mean	53.24 (8.33)	63.1 (5.87)	48.72 (8.63)	45.79 (6.27)	
Physical activity	1.77	−0.34	−0.31	−0.36	833.81*
Mean (MET/week)	71.11 (23.55)	16.18 (13.69)	16.89 (17.54)	15.51 (13.79)	
Media use	−0.14	−0.39	1.52	−0.41	643.81*
Mean (h/day)	2.85 (1.75)	2.29 (1.30)	6.56 (2.14)	2.24 (1.08)	

*p < .001.

HuSKY = healthy nutrition score.

#### Anthropometric measures

Height was measured with a portable telescopic height measuring scale (SECA, Hamburg, Germany; accuracy: 0.1 cm) with the participants standing upright without shoes. Body mass was measured with an electronic scale (SECA, Hamburg, Germany; accuracy: 0.1 kg), while participants were asked to take shoes and heavy clothes off. The measurements were performed by skilled test leaders, who were periodically trained. BMI was calculated as body mass divided by height squared (kg/m^2^). International age- and gender-specific cut points [53] were used to classify participants into normal weight or overweight. In this study, the term overweight includes overweight and obese subjects.

#### Self-rated health

SRH was measured with a single item because there is consistent evidence that SRH as a single item is a valid measure for general health [20]. Participants were asked in the KiGGS Survey (T1: questionnaire; T2: telephone interview) how they would rate their state of health in general. Answer categories were “very good”, “good”, “fair”, “poor” and “very poor”. These possible answers were coded from 1 (=very good) to 5 (=very poor). Longitudinal studies showed that this scale provides stable results on the construct of SRH during adolescence [54, 55].

### Participants

Of the 1,642 participants included in the cluster analysis at T1, 556 participants attended the physical examination at T2 and hence had longitudinal data on weight status. Data of this subsample was used for further analysis on weight status. This study population consisted of 283 female and 273 male participants (50.9% and 49.1%, respectively) between 11 and 17 years at T1 and between 17 and 24 years at T2 (mean age at T1: 13.5 ± 2.0 years; mean age at T2 20.2 ± 2.0 years). This longitudinal sample did not differ in the socio-structural variables age and sex from the remaining subjects from T1, but socio-economic status (SES) and migration background differed significantly between these two groups (see [29] for the description of their measurement). In the longitudinal sample, 21.4% had a low, 51.7% a medium and 26.7% a high SES. In the group of subjects with only T1 data, 27.7% had a low, 49.9% a medium and 22.4% a high SES. 7.2% of the longitudinal sample and 11.0% of the T1 only sample had a migration background.

Data on SRH at T1 and T2 were available for 953 participants (54.5% female, 45.5% male) aged 11 to 17 years at T1 and 17 to 24 years at T2 (mean age at T1: 14.1 ± 1.9 years; mean age at T2: 20.1 ± 1.9 years). This longitudinal sample did not differ in age and migration background from the remaining subjects from T1, but the socio-demographic items sex and SES differed significantly between these two groups. 42.2% of subjects with T1 data only were female and 57.8% were male. In the longitudinal sample, 21.3% had a low, 52.6% a medium and 26.1% a high SES. In the group of subjects with only T1 data, 31.4% had a low, 47.6% a medium and 20.9% a high SES.

### Statistical analyses

All statistical tests were performed in SPSS statistical software for Windows Version 21.0 (IBM Corporation, Armonk, NY, USA). McNemar Tests were used to reveal significant differences of prevalence of overweight between T1 and T2 in the four clusters as well as for subgroups. With subgroups smaller than N = 30 Yates correction (0.5) was used. Multinomial logistic regression analysis was used to calculate the odd’s ratio (OR) (95% confidence interval (95% CI)) of changing weight status dependent on cluster membership. Age, sex, socio-economic status and cluster membership were included in the model. To reveal significant differences of mean SRH between T1 and T2 T-tests for paired samples were used. Two-way ANOVA with repeated measures (within subject factor: time) were performed to analyze differences in terms of change of SRH between clusters. The significance level for all statistical tests was set a priori to *α* = 0.05.

## Results

### Associations of health-related behavior patterns with weight status change

In all clusters the percentage of overweight members increased from T1 to T2 (Table 2). This increase was statistically significant for clusters 2, 3 and 4 but not for cluster 1 (high physical activity level). The increase was greatest in cluster 3 (very high media use), which also had by far the highest percentage of overweight subjects.

**Table 2:** **Percentage of overweight members of the clusters for T1 and T2**

Cluster	N	T1	T2	T2-T1	McNemar Chi ^2^	p
Cluster 1	87	19.5%	21.8%	+ 2.3%	0.5	.480
Cluster 2	210	14.3%	20.5%	+ 6.2%	5.12	.024
Cluster 3	89	24.7%	39.3%	+ 14.7%	8.05	.004
Cluster 4	170	14.1%	21.2%	+ 7.2%	6.55	.011
Total	556	16.7%	23.9%	+ 7.2%	19.05	< .001

Clusters 2 (high healthy nutrition score) and 4 (low scores on all included indices) had a significant increase of overweight in female subjects (Table 3). For male subjects, cluster 3 had a significant change in weight status and was the subgroup with the largest increase of overweight members.

**Table 3:** **Percentage of overweight members of the clusters for T1 and T2 for each sex separately**

Cluster	Sex	N	T1	T2	T2-T1	McNemar Chi ^2^	p
Cluster 1	male	59	18.6%	20.3%	+ 1.9%	0.14	.705
	female	28	21.4%	25.0%	+ 3.6%	0.25	.617
Cluster 2	male	87	13.8%	18.4%	+ 4.6%	1.14	.285
	female	123	14.6%	22.0%	+ 7.4%	4.26	.039
Cluster 3	male	61	26.2%	44.3%	+ 18.1%	7.12	.008
	female	28	21.4%	28.6%	+ 7.2%	0.56	.453
Cluster 4	male	66	15.2%	21.2%	+ 6.0%	1.60	.206
	female	104	13.5%	21.2%	+ 6.7%	5.33	.021
Total	male	273	18.0%	25.3%	+ 7.3%	8.33	.004
	female	283	15.6%	22.6%	+ 7.0%	11.11	< .001

While the older age groups in all clusters – except in cluster 1 – showed a significant increase in overweight members (Table 4), no significant change was observed for the younger age groups. In cluster 3, the absolute difference in change in weight status over time between the younger and the older members was the greatest.

**Table 4:** **Percentage of overweight members of the clusters for T1 and T2 for two age groups separately**

Cluster	Age group T1 (yrs)	N	T1	T2	T2-T1	McNemar Chi ^2^	p
Cluster 1	11-13	48	18.8%	20.8%	+ 2.0%	0.20	.655
	14-17	39	20.5%	23.1%	+ 1.6%	0.33	.564
Cluster 2	11-13	125	12.8%	16.8%	+ 4.0%	1.47	.225
	14-17	85	16.5%	25.9%	+ 9.4%	4.00	.046
Cluster 3	11-13	35	17.1%	22.9%	+ 5.8%	0.67	.414
	14-17	54	29.6%	50.0%	+ 20.4%	8.07	.005
Cluster 4	11-13	96	15.6%	17.7%	+ 2.1%	0.40	.527
	14-17	74	12.2%	25.7%	+ 13.5%	8.33	.004
Total	11-13	304	15.1%	18.2%	+ 3.1%	2.63	.105
	14-17	252	18.6%	30.6%	+ 12.0%	19.57	< .001

Multinomial logistic regression analysis showed that over all cluster membership had no significant impact on changing weight status, but age (p = .002) and SES (p = .003) were significant predictors for changing weight status. With regard to the group of subjects who changed from normal weight at T1 to overweight at T2 (vs. normal weight at T1 and normal weight at T2) it was shown that members of cluster 3 were more likely to change from normal weight to overweight over the period of six years (OR: 3.491; 95% CI: 1.178-10.346; p = .024; reference category: cluster 1). No significant results were found for clusters 2 and 4.

### Associations of health-related behavior patterns with change in SRH

SRH improved from T1 to T2 in clusters 1, 2 and 3 (Table 5), but these changes were not statistically significant. In cluster 4, mean SRH remained the same over time. The greatest improvement was observed in cluster 1 (high physical activity level).

**Table 5:** **Mean (SD) self-rated health in the clusters for T1 and T2**

Cluster	N	T1	T2	T2-T1	T	df	p
**Cluster 1**	151	1.78 (0.64)	1.66 (0.65)	- 0.12	1.94	150	.055
**Cluster 2**	339	1.86 (0.55)	1.82 (0.58)	- 0.04	1.12	338	.263
**Cluster 3**	153	2.01 (0.65)	1.94 (0.67)	- 0.07	1.18	152	.240
**Cluster 4**	310	1.85 (0.58)	1.85 (0.59)	0	- 0.24	309	.808
**Total**	953	1.87 (0.59)	1.82 (0.62)	- 0.05	1.82	952	.068

SRH improved significantly in all male participants but not in female participants (Table 6). While SRH improved in the male subgroup in cluster 1, SRH did not change significantly in male members of clusters 2, 3 and 4 and in none of the female subgroups. In all subgroups separated by age SRH did not change significantly with the exception of the older group of cluster 1, where a significant improvement of SRH was found (Table 7).

**Table 6:** **Mean (SD) self-rated health in the clusters for T1 and T2 for each sex separately**

Cluster	Sex	N	T1	T2	T2-T1	T	df	p
Cluster 1	male	106	1.81 (0.69)	1.58 (0.63)	- 0.23	3.12	105	.002
	female	45	1.71 (0.51)	1.84 (0.67)	+ 0.13	- 1.23	44	.225
Cluster 2	male	119	1.82 (0.57)	1.76 (0.56)	- 0.06	.80	118	.425
	female	220	1.88 (0.54)	1.85 (0.58)	- 0.03	.80	219	.425
Cluster 3	male	90	1.89 (0.66)	1.83 (0.64)	- 0.06	.69	89	.495
	female	63	2.19 (0.59)	2.10 (0.69)	- 0.09	1.03	62	.307
Cluster 4	male	119	1.82 (0.57)	1.84 (0.58)	+ 0.02	- .39	118	.698
	female	191	1.86 (0.59)	1.86 (0.60)	0	0	190	1.00
Total	male	434	1.83 (0.62)	1.76 (0.61)	- 0.07	2.11	433	.035
	female	519	1.90 (0.57)	1.88 (0.62)	- 0.02	.51	518	.613

**Table 7:** **Mean (SD) self-rated health in the clusters for T1 and T2 for two age groups separately**

Cluster	Age group T1 (yrs)	N	T1	T2	T2-T1	T	df	p
Cluster 1	11-13	56	1.64 (0.55)	1.70 (0.63)	+ 0.06	- .54	55	.595
	14-17	95	1.86 (0.68)	1.64 (0.67)	- 0.22	2.89	94	.005
Cluster 2	11-13	136	1.81 (0.59)	1.84 (0.61)	+ 0.03	- .50	135	.619
	14-17	203	1.89 (0.52)	1.80 (0.56)	- 0.09	1.89	202	.060
Cluster 3	11-13	44	1.93 (0.63)	1.86 (0.59)	- 0.07	.65	43	.519
	14-17	109	2.05 (0.66)	1.97 (0.70)	- 0.08	.99	108	.327
Cluster 4	11-13	124	1.90 (0.58)	1.79 (0.51)	- 0.11	1.74	123	.085
	14-17	186	1.81 (0.58)	1.90 (0.64)	+ 0.09	−1.86	185	.065
Total	11-13	360	1.83 (0.59)	1.80 (0.58)	- 0.03	.74	359	.459
	14-17	593	1.89 (0.60)	1.84 (0.64)	- 0.05	1.74	592	.083

ANOVA with repeated measurements revealed no significant differences in change in SRH between clusters.

## Discussion

The purpose of this study was to define high-risk patterns of health limitations and to obtain insights into the complex structure of multiple health behaviors. We examined the longitudinal association of health-related behavior patterns in adolescents in Germany with change in (a) weight status and (b) SRH and (c) stratified the data by sex and age group. Different health-related behavior patterns led to different changes in weight status and SRH and these differences were sex- and age specific.

### Change in weight status

The percentage of overweight persons increased in all four health-related behavior clusters. This result is not surprising because the prevalence of overweight in Germany increases from adolescence to around 60% in adulthood [56]. The behavior pattern of cluster 1 (high physical activity level, average diet quality and media use) appears to be the most protective behavior pattern for developing overweight. Results of the few studies which analyzed longitudinal associations of behavior patterns with overweight [24, 37–39] can only be partly compared to the results found in the present study, as the included variables differed between the studies. However, some consistencies can be stated: Landsberg et al. [39] found the lowest incidence of obesity in their cluster which combined a high physical activity level with low media time (amongst other factors). Further, in the study of Boone-Heinonen et al. [24] the lowest incidence of obesity was shown in the “school clubs and sports” cluster (while in their study significant differences where only found in girls’ clusters). These results support the assumption, that a high physical activity level may have a protective effect on weight gain. In contrast, the combination of high media use, low physical activity level and low diet quality (cluster 3) appears to carry the greatest risk for gaining weight, which is in agreement with results of previous studies focusing on these individual behaviors. There is evidence that physical inactivity [1], poor diet quality [2] and high media use time [57] are associated with a higher risk of being overweight. In addition, Gubbels et al. [37] found the highest incidence of obesity in their “sedentary-snacking” cluster, which highlights the role of sedentary behavior/media use in terms of weight gain.

Membership in cluster 3 was associated with a higher chance of changing weight status from normal weight to overweight over the period of six years compared to membership in cluster 1. In clusters 2 and 4 the chance of becoming overweight was not significantly different to the reference cluster 1. Hence, in this study only members of cluster 3 could have been shown to be a risk-group for becoming overweight. The significant increases in overweight prevalence in clusters 2 and 4 might be explained by the influence of age and SES, as multinomial logistic regression implied. These results further indicate that a low physical activity level per se does not seem to increase the risk of becoming overweight, as physical activity levels in cluster 2, 3 and 4 were similar. In contrast, Landsberg et al. [39] conclude from their results “that low activity plays a major role in the development of childhood obesity”. Further longitudinal studies (with higher sample sizes) are needed to clarify the role of physical activity as well as the risk potential of behavior patterns such as those of clusters 2 and 4, which combine health-enhancing as well as health-compromising behaviors and therefore cannot be titled as “healthy” or “unhealthy” that easily.

The prevalence of overweight at T1 and T2 must be interpreted carefully because of selection effects of the longitudinal sample. The proportion of overweight members in the clusters at T1 in this study differed from to that shown in a previous study [29]. Nonetheless, the results suggest that cluster 3 is a high-risk group for overweight compared to the other health-related behavior clusters. Moreover, the extremely higher percentage of overweight males than females in this cluster suggest that – at least in this sample – especially men with the behavior pattern of cluster 3 are at high risk of becoming overweight. This may be associated with the fact that boys tend to have higher media use than girls [58]. Further analyses of our data confirmed that in cluster 3 boys had a significantly higher media use than girls (6.5 hours vs. 5.5 hours/day). In addition, girls are known to engage more than boys in other activities which contribute to energy balance such as meeting up with friends and shopping [59] (which were not included in the physical activity index). This may be an additional reason for the smaller increase in overweight prevalence in girls than in boys of the third cluster. However, Boone-Heinonen [24] found significant incidences of obesity only in female clusters, which contrasts our results and emphasizes the need of further research in this field.

Another finding of this study is that in all clusters besides cluster 1, the older age groups showed a significant increase in overweight prevalence while the younger age groups did not. First, this result underlines the assumption that solely the behavior pattern of the first cluster seems to be able to protect overweight. In the older age group of cluster 3 the increase was substantially the highest, what emphasizes the high risk of weight gain with a behavior pattern of low physical activity level, low diet quality and high media use especially in older adolescents. Second, several possible reasons should be discussed for the appearance of significant increases in overweight prevalence only in older but not younger age groups. One possible reason might be that growth and maturation influenced longitudinal change of BMI especially in younger adolescents. As during early adolescence body height and weight are rapidly changing, adolescents must necessarily be in a slight positive energy balance due to physiological demands of growth and maturation [60]. The slight positive energy balance in the younger age group at T1 might be a reason for the fact that overweight prevalence did not increase significantly in younger adolescents. The assumption is supported by the slightly higher overweight prevalence in 11–13 year old adolescents in comparison to 14–17 year olds in a large representative sample for Germany [61]. Further, behavior patterns potentially might not jet be as stable in younger adolescents as in the older group. Craigie et al. [5] found in their review that tracking of physical activity was greater with increasing age at baseline assessment. This might be one reason why no association of behavior patterns with change of overweight prevalence could have been detected. Further research on the stability of cluster membership over time is needed to answer this question.

### Change in SRH

SRH did not change significantly over time in any of the four behavior clusters. This result is in agreement with Breidablik et al. [54] who found that only a small percentage of adolescents reported a major change in their rating of subjective health over four years. Although not statistically significant, a trend of improved SRH was observed, especially in cluster 1. This tendency may be explained by the fact that adolescence is a time of large changes in life, which may negatively influence the rating of SRH in adolescents but not in young adults. Further, results indicated that values in SRH at T2 were significantly lowest (=best SRH) in cluster 1. Studies showed that physical activity is positively associated with better health-related quality of life [62] and can be related to improved psychological and social functioning [63, 64]. This may lead to a more positive subjective rating of health in cluster 1. High physical activity levels have previously been associated with better SRH in adolescents [65, 66], and high media use time (≥four hours and 15 minutes) has been associated with poorer SRH [67]. These associations may explain the poorest self-rating of health at T2 in cluster 3 (high media use, low physical activity level and low diet quality).

Only male members of cluster 1 reported significantly improved SRH at T2 compared to T1. Overall, male participants had a small but significant improvement in SRH over time. In comparison, Breidablik et al. [54] showed that more adolescent girls than boys report a decline of SRH. Thus, both studies showed that change of SRH over time tends to be rather positive in boys than in girls. However, the positive tendency was stronger in the present study. One reason might be that SRH was assessed by telephone interview at T2 versus paper-pencil questionnaire at T1. The tendency to rather positive answer categories is higher in interviews than in written questionnaires [68, 69].

### Implications of the obtained results

While the prevalence of overweight increased, SRH showed the tendency to slightly improve (even though this change was not significant) or at least stay at the same level in all four health-related behavior clusters. This difference may be explained by the different trends of weight status and SRH across lifespan. The prevalence of overweight is lower in childhood [70] than in adulthood [56]. However, Wade et al. [71] reported a decline in SRH from grade 7 to grade 9 to grade 11 and a slight increase (in boys) or no change (in girls) from grade 11 to 13. Hence, SRH appears to plateau in young adulthood, which might be explained by the end of puberty or – as suggested by Wade et al. [71] – reflect a stabilization of perceptions of SRH. Thus the results of the present study seem to comply with normal development of weight status and SRH.

Overall, of the four identified clusters [29], cluster 3 (low diet quality, low activity level, high media use) can be termed the high-risk cluster. Especially male members as well as the older age group of this cluster seem to have the highest risk of incurring health limitations. In contrast, persons in cluster 1 (averaged diet quality and media use, high physical activity level) seem to be protected best from health limitations.

The results of this study emphasize that Multiple Health Behavior Research can be used for clustering health-related behaviors and discovering cumulative and compensatory effects of health-related behaviors on each other. Based on the results of this study, it can be assumed that:

- High media use in combination with a low physical activity level and poor diet quality is associated with a relevant risk of health limitations. While a meta-analysis of Marshall et al. [57] revealed only small relationships of media use time and body fatness with arguable clinical relevance, the results of the present study indicate a relevant association. Marshall et al. [57] reported that 8 of the 51 included studies were longitudinal studies and that mean effect size did not differ significantly between longitudinal and cross-sectional studies. Hence, the greater association between high media use, low physical activity level and poor diet quality found in the present study may primarily be based on the combined examination of different health behaviors and strengthens the assumption of Marshall et al. [57] that “possible relationships may be confounded by other factors such as the consumption of energy-dense snacks that may accompany these behaviors”.
- Fairly high media use (two hours and 50 minutes in cluster 1) might be compensated by a high physical activity level and averaged diet quality and hence not result in health limitations.
- In younger adolescents, the combination of a low activity level and poor diet quality only seems to be associated with a high risk of health limitations when combined with high media use. The question arises why low media use seems to protect young adolescents from becoming overweight because media use per se does not account for energy-balance. One possible explanation for this association is that low media use may be related to a greater physical activity in everyday life (which was not included in the physical activity index in this study) and hence account for a higher energy use. However, in late adolescence a higher risk of health limitations in this pattern seems to develop.

### Limitations

The current study was based on a previous cluster analysis of data collected via self-administered questionnaires. Statements on diet behavior can be affected by the subjective rating of portion sizes and by the difficulty of recalling the frequency and amount of food intake. Moreover, statements on physical activity level and media use can be affected by the difficulty of remembering the duration of activities and summarizing this information. The choice of collecting data by questionnaire was predetermined by the size of the survey population. The indices used in this study do not provide detailed insights into different aspects of the health-related behaviors but were adequate for providing an overall estimate of health-related behavior patterns. It can be assumed, that these patterns are valid at least for Germany [29] and therefore built a foundation for the present investigation.

The longitudinal data used in this study was selective. As outlined in the methods section, the non-respondents at T2 differed in terms of socio-demographic variables from the respondents which is a common problem with longitudinal data [72]. Consequently, the results cannot be readily generalized. Moreover, in comparison to baseline results on cluster characteristics [29], coincidental selection effects could have been discovered. This fact limited the interpretation of levels of weight status and SRH. Nonetheless, this study provides information on the change of health parameters in a large longitudinal sample and identified high-risk patterns of health-related behavior. Further investigations should attend to the question, if behavior patterns are indeed stable over time and if switching patterns is common. Hence it would be possible to investigate the benefit of lifestyle change.

## Conclusions

This study provides information on the change of health parameters in adolescents and young adults and identified high-risk patterns of health-related behavior. Further, identifying cumulative as well as compensatory effects of different health-related behaviors on each other emphasizes the importance of Multiple Health Behavior Research. The information gained in this study contributes to a better understanding of the complexity of health-related behavior and its impact on health parameters. Identifying high-risk patterns is critical for designing prevention programs specifically targeted at high-risk groups.
